# Growing use of valproic acid in substance use disorders

**DOI:** 10.1192/j.eurpsy.2022.628

**Published:** 2022-09-01

**Authors:** J. Romão, M. Gonçalves, M. Ribeiro, R. André, R. Saraiva, M. Abreu

**Affiliations:** 1 Centro Hospitalar Universitário Lisboa Norte, Psychiatry, Lisboa, Portugal; 2 Centro Hospitalar Lisboa Norte, Psychiatry, Lisboa, Portugal

**Keywords:** substance use disorder, cocaine, valproate

## Abstract

**Introduction:**

Valproic acid is an antiepileptic drug used in different fields of Psychiatry. It is known mostly for its use in managing patients with bipolar affective disorder. In psychiatry of addiction, there is still no approved indications for its usage, but it is widely prescribed in treating alcohol and cocaine abuse, due to the existence of studies in these addictions.

**Objectives:**

This review aims to clarify the relation between valproic acid and dependences, particularly cocaine.

**Methods:**

Non-systematic literature review using a PubMed search, using the following key words: “valproate”; “cocaine use”.

**Results:**

Cocaine dependence can decrease GABA levels in humans. Valproic acid has multiple mechanisms that favour the synthesis of GABA, potentiating its release and postsynaptic GABAergic response. Because of this, valproic acid was found effective in promoting abstinence and in reducing the use of cocaine. There are studies that support the valproic acid’s use in alcohol and cocaine dependences. Valproic acid has been shown to be promising in relapse prevention. It has also showed efficacy in the management of impulsivity and irritability, what makes it useful in managing patients with borderline personality disorder – patients at higher risk for alcohol or substance use disorders.

**Conclusions:**

Cocaine addiction involves different phenomena and may respond to distinct pharmacologic approaches. Although some studies need to be confirmed by larger clinical trials, valproic acid seems a promising agent as one of some potential treatments for cocaine dependence. Further studies are required in this field to come to more reliable conclusions.

**Disclosure:**

No significant relationships.

